# Disturbance of Ecological Self and Impairment of Affordance Perception

**DOI:** 10.3389/fpsyg.2022.925359

**Published:** 2022-06-06

**Authors:** Nam-Gyoon Kim, Judith A. Effken

**Affiliations:** ^1^Department of Psychology, Keimyung University, Daegu, South Korea; ^2^College of Nursing, University of Arizona, Tucson, AZ, United States

**Keywords:** affordance, disturbance of minimal self, schizophrenia, Alzheimer’s disease, contra-positive evidence, ecological self, empirical investigation of meaning

## Abstract

Affordance, a radical concept James Gibson introduced in the 1970s, remains controversial today. Defined as environmental properties taken with reference to an animal’s anatomy and action capabilities, affordances are opportunities for action the environment offers. By perceiving affordances, organisms hold meaningful relationships with their surroundings. Affordance is not just a theoretical concept but, as the embodiment of meanings and values, has serious psychological implications. We contend that the lack of these meanings and values underlies the irrational behavior seen in patients with self disorders such as schizophrenia. We reason that it is by perceiving affordances that individuals keep in touch with their surroundings and stay mentally healthy. Using contrapositive reasoning, the reverse could also be true. That is, when individuals experience difficulty maintaining meaningful relations with their surroundings and suffer from mental health problems, we might anticipate that their affordance detection systems are impaired. In two studies conducted in our laboratory, patients with schizophrenia and Alzheimer’s disease were shown to have impaired capacity to perceive affordances, a result qualifying as contra-positive evidence corroborating the affordance concept. In addition, our results provide support for accepting contra-positive evidence as a complementary tool to positive evidence for empirically validating concepts such as affordance and meaning.

## Introduction

Of the many ideas put forth by the American perceptual psychologist James Gibson, *affordance*, without question, is the most radical (Gibson, 1977, 1979, 1982). The conventional account of how an animal interacts with the surrounding environment begins with physical energies impinging on sensory receptors. When stimulated, the receptors transduce the energies into neural signals that spread across cortical and subcortical regions while undergoing several stages of enrichment. Such elaboration processes are mandated because the impoverished sensory input is devoid of meaning (having been produced by the meaningless physical entities comprising the environment) and therefore cannot represent the surroundings adequately. However, animals routinely interact with the environment (i.e., with objects, places, events, and other animals) in meaningful ways.

Gibson rejected a dualistic stance that separates an (objective) physical world devoid of meanings from (subjective) mental states replete with meanings. Instead, Gibson envisioned that animal and environment are reciprocally conjoined, thus forming an inseparable pair. To portray the reciprocal relationship of animal and environment, Gibson defined the properties of the environment, not in units of pounds, feet, and seconds, but in units of the animal’s body dimensions and action capabilities, i.e., affordances (Warren, 1984; Mark, 1987). With its properties defined as affordances, the environment now abounds with behavior-relevant properties that offer opportunities for animals to act. Moreover, with environmental properties expressed in animal-referential terms, its descriptors now are compatible with those of the animal, enabling the animal’s contact with the environment to be direct, epistemic, and meaningful. As Gibson (1979) put it, “If what we perceived were the entities of physics and mathematics, meanings would have to be imposed on them. But if what we perceive are the entities of environmental science, their meanings can be discovered” (p. 33).

Suppose that a human animal has a specific goal in mind. It actively searches its surroundings for an affordance that can help it reach its goal. When a relevant affordance is identified, the human animal uses that affordance to implement and fine-tune its actions until its goal is realized. Subserved by an affordance, this encounter with the environment yields a unique experience. Each experience, however insignificant or trivial, involves the combined effort of the perception-action system. Significantly, whether it is reaching out and grasping a mug or pulling a chair and sitting on it, successful accomplishment of an intended action is a manifestation of autonomy and control, assuring one’s sense of agency. This may be why each experience serving as the embodiment of meaning motivates the animal to continue interacting with its surroundings.

What might transpire if the human animal’s capacity to access affordances is somehow compromised? No longer able to appreciate affordances or maintain meaningful relationships with others or with its surroundings, the consequences for the animal would be dire. This is likely the case with some of the most devastating mental disorders, particularly those disorders phenomenological psychopathologists suspect to arise from disturbance of the minimal self (i.e., self-disorder). As their ability to register affordances decreases, these human animals become increasingly deprived of meanings and values, eventually developing severe mental health symptoms. Based on this reasoning, we propose and demonstrate the use of an indirect way to validate the concept of affordance. We further propose utilizing patients with mental disorders suspected to be caused by disturbance of minimal self as a testing ground to validate the concept of affordance, and at the same time, provide an empirical investigation of meaning.

In contemporary cognitive science, “people disappear and are replaced by symbolic constructs and manipulations analogous to those of computer programs” (Reed, 1996, p. 3). Gibson and his followers have rejected this approach by restoring the experiences and activities of persons and animals to psychological reality. A similar sentiment has been raised by Varela et al. (1991) who noted that “cognitive science has had virtually nothing to say about what it means to be human in everyday, lived situations” (p. xv). The philosophical perspective put forth by Varela and his followers is known as enactivism [Varela et al. (1991); Noë (2004), and de Haan (2020), for review]. Since enactivism and ecological psychology both denounce mental representation to account for cognition, several members of ecological camp have considered whether an integrated conceptual framework is possible. Thus far, all have found these efforts futile [see Flament-Fultot et al. (2016); Heft (2020), and Read and Szokolszky (2020)]. The irreconcilability between the two perspectives lies in the way sensation is conceived. Further discussion of these philosophical positions and their differences is beyond the scope of the present study so we suggest that the reader refer to the cited references for further details.

In the following, we further delineate the concepts of affordance and ecological self and then describe our own psychophysical studies assessing affordance perception capacity.

## Affordances

A hiker went for a long hike in Central Park in New York City. After several hours, the hiker encountered a horizontal, flat, extended, and rigid surface at approximately knee height. Whether that surface is a park bench, a tree stump, or a swing, it offers an opportunity for the hiker to sit and rest. However, flat, extended, and rigid surfaces provide places to sit only for those individuals whose lower leg length corresponds roughly to the height of the seats.

Clearly, a sit-on-able surface for an adult is different from that for a 2-year old, but both offer sit-on-able affordances. Thus, a sit-on-able affordance exists, irrespective of an individual’s age and/or physical makeup. In this sense, affordances are objective properties. However, a sit-on-able surface is uniquely tailored, not only for an individual’s specific body dimensions, but also for that individual’s specific needs and circumstances. For example, for someone with a painful, swollen hip, sit-on-able is not an affordance a hard park bench offers. Thus, affordances are also subjective. As Gibson (1979) noted, “an affordance is neither an objective property nor a subjective property; or it is both… (that is, it) cuts across the dichotomy of subjective—objective” (p. 129).

When defined in reference to an individual’s action capabilities, the environment offers many opportunities for the individual to act. As was the case with the hiker described above, an individual perceives an affordance that would fulfill his needs at a given moment by detecting the information specifying that affordance. Visually, the ambient light structure at an observation point is uniquely determined by the composition and layout of the surrounding surfaces and is specific to the affordances those surfaces offer. For example, rigid surfaces engender patterns that differ from those of elastic surfaces (von Fieandt and Gibson, 1959). Although the number of surfaces comprising our surroundings is infinite and of many types, the number of dimensions along which surfaces can vary is finite, thus limiting the number of optical invariants to which an organism must attune.

To exploit the available environmental information about a sit-on-able surface, our hiker had to seek it actively. In the words of Gibson (1979), “We must perceive in order to move, but we must also move in order to perceive” (p. 223). In the case of vision, this involves not only using the eyes, but “the eyes in the head on the shoulders of a body that gets about” (Gibson, 1979, p. 222). The information specifying an affordance must be actively detected by our hiker, but the hiker’s action is guided by perceptual information. As the hiker moves, the ambient energy distribution is uniquely transformed in accordance with the changes in the environmental layout and the displacements of the observation point. This transforming energy pattern at a moving point of observation (i.e., optic flow) is specific both to the environmental layout and the animal’s movements that engendered it (Gibson, 1979; Warren, 1998, 2006, 2021). Our hiker’s forward movement structured the optic flow such that all optical elements radiated from a single point (i.e., the focus of expansion) corresponding to the hiker’s own movement direction. By regulating the direction of movement coincident with the focus of expansion, our hiker was able to reach the intended target, then sit down and rest.

Although the structured light is ambient about an observation point, our hiker could sample only a portion of the optic array due to a limited field of view [see Figure 7.1 in Gibson (1979), p. 113]. That field of view, if portrayed as an oval window, contained various optical structures, some of which corresponded to the hiker’s body parts (e.g., orbits of the eyes, nose, upper lip, cheeks, and limbs). As the hiker moved (e.g., turning from side to side), those optical structures corresponding to body parts transformed. Because the optical transformation was produced by the hiker’s movement, the transformation patterns were specific to those movements. For Gibson, perception of the environment and perception of the self were inseparable, always occurring together (Reed, 1996). As Gibson (1979) remarked, “One perceives the environment and co-perceives oneself” (p. 126).

In the optic array, information specific to the environment is called extero-specific and information specific to the observer is called proprio-specific. Since, Sherrington (1906), it has been thought that self-perception is conveyed by information from mechanoreceptors in the muscles, tendons, and joints. For Gibson, however, awareness of self can also be gained visually (e.g., as *visual kinesthesis)*.

“Vision is *kinesthetic* in that it registers movements of the body just as much as does the muscle-joint-skin system and the inner ear system. … Visual kinesthesis goes along with muscular kinesthesis. The doctrine that vision is exteroceptive, that it obtains ‘external’ information only, is simply false. Vision obtains information about *both* the environment and the self” (Gibson, 1979, p. 183, italics original).

Gibson (1977, 1979, 1982) defined a set of affordances as an *ecological niche*. All living organisms are equipped with the capacity to perceive affordances, which enables them to exploit the myriad of affordances the surrounding environment offers. However, if one’s capacity to perceive affordances has been compromised, the consequences are likely to be devastating because the environment has ceased to be meaningful. This appears to be the case in patients with certain clinical and mental disorders [e.g., schizophrenia, post-traumatic stress disorder (PTSD), and Alzheimer’s disease (AD)]. A common feature of these disorders is a disturbance of what might be called, as Gallagher (2000, p. 15) did: the “basic, immediate, or primitive ‘something’ that we are willing to call a self.”

## Ecological Self

As noted earlier, optic flow is determined by facts about the environment and facts about the observer. Optic flow structure, therefore, can be decomposed into two components, one determined by the environment and the other by the observer. When an observer moves, the entire flow field is disturbed (a global transformation); but when an object in the environment moves, it perturbs the flow field locally (a local transformation) (Fajen and Kim, 2002). As an observer moves, producing a global transformation in the optic flow, the observer is immediately aware of causing this transformation. Neisser (1988) referred to such self-specification in optic flow as “ecological self.” As an active agent in the immediate environment, the ecological self “perceives themselves, among other things: where they are, how they are moving, what they are doing, and what they might do, whether a given action is their own or not” (Neisser, 1993, p. 4).

Ecological self can be understood as what phenomenological philosophers call “ipseity” (ipse in Latin meaning “self” or “itself”), also called minimal self, core self, or proto self. The minimal self is characterized by two separable modalities of pre-reflective and primitive subjective awareness, that is, a sense of ownership (awareness of being the source of phenomenal experiences) and a sense of agency (awareness of being the agent executing one’s own actions) (Gallagher, 2000; Sass and Parnas, 2003; Zahavi, 2005; Stanghellini, 2009; Fuchs, 2010; Parnas and Sass, 2010; Nelson et al., 2014).

Given its diverse (e.g., positive, negative, and disorganized) mental symptoms, schizophrenia is arguably the most debilitating, yet the most perplexing, of all mental disorders (Arango and Carpenter, 2011). Currently, a schizophrenia diagnosis is based on criteria defined in the American Psychiatric Association’s Diagnostic and Statistical Manual of Mental Disorder (DSM-5) and the World Health Organization’s International Statistical Classification of Diseases and Related Health Problems (ICD-10), as well as on structured interviews. The present diagnostic system aims to identify the underlying cognitive and neurobiological processes for each symptom or symptom group comprising schizophrenia’s psychopathology (Persons, 1986; Cahill and Frith, 1996). Although recent progress in neuroscience and molecular genetics has furthered our understanding of this disability [see Weinberger and Harrison (2011), for a review], the exact cause of this disease remains elusive (Wong and Van Tol, 2003; Insel, 2010; Jablensky, 2010).

Recently, proponents of phenomenological psychiatry and philosophy have underscored the subjective experience of the patient as a valuable tool for gaining an in-depth understanding of the disorder. These researchers suggest that the disparate psychopathological symptoms of schizophrenia may actually be manifestations of a single phenomenological core: *disturbance of ipseity*. The sources of this self-distortion are thought to be two mutually interdependent processes—hyper-reflexivity and diminished self-presence. Hyper-reflexivity refers to an intensified self-consciousness directing the patient’s focal attention to internal feelings; while diminished self-presence refers to a weakening sense of self existence (Sass and Parnas, 2003).

Individuals’ awareness of their own thoughts, actions, perceptions, feelings, or pain operates at a pre-reflective (i.e., direct, immediate, implicit, or non-conceptual) level. With alterations of self, patients may feel their presence in this world diminish and their grip on the world slip away, or feel that they are falling under the control of an alien. Their perceived presence disintegrates, receding into the background; and the boundary separating their perceived selves from others vanishes (Parnas, 2000, 2003, 2012; Sass, 2003a,b, 2014; Sass and Parnas, 2003; Cermolacce et al., 2007; Parnas and Sass, 2010; Raballo et al., 2011; Nelson et al., 2014). Simultaneously, an opposite process is underway. The patients’ own bodies begin to feel strange and unfamiliar, inviting their explicit attention. As their self-monitoring increases, the surrounding world no longer draws their attention. Gradually their focus of attention shifts inward to reflect on their own mental activity. As self-directed reflection intensifies, aspects of their awareness may separate or detach as if they were external objects. As their selves become more alienated from their bodies and aspects of their own feelings, their actions and expressions no longer feel natural and may result in delusions of alien influence (Fuchs, 2009, 2013).

Phenomenological psychopathologists conceptualize schizophrenia as a disorder triggered by disturbance of minimal self that alienates self from the body and from the world, as depicted above. For Gibson (1979), affordance links the environment and the observer as an inseparable dual: “the awareness of the world and one’s complementary relations to the world are not separable” (p. 141). If affordance is what connects the self and the world (i.e., animal and the environment), then the disruption of affordances, for example, due to an impairment in the capacity to perceive them would be expected to separate the self and the world. With impaired affordance perception capacity, patients would increasingly fail to register affordances. As their affordance capacity further deteriorates, their surroundings, once replete with values and meaning, ultimately becomes a barren field. Entrapped in meaningless surroundings, the patients can no longer maintain meaningful relations with environmental entities so retreat from social interactions and other activities, gradually disconnecting from reality until they take on the characteristics of a soulless body or a disembodied spirit (Stanghellini, 2009).

## Psychophysical Investigation of Affordance Perception Capacity

Human artifacts are designed with specific functions in mind. However, these artifacts often provide more than one affordance owing to their multiple properties (e.g., shape, size, material composition, etc.). When a specific tool is not available, we often use other household items to carry out functions beyond those for which they were originally designed if the items provide affordances subserving our intended goal. For example, lacking a screwdriver, we might instead use a coin to drive a screw. Flatness and rigidity, two properties of a coin, provide a “drive-a-screw-able” affordance.

A set of diverse objects, each with a different primary affordance, can offer the same secondary affordance. For example, a bowl or, a jam jar, can be used to collect water from the faucet but so can a shoe or a safety helmet. Kim et al. (2022) used a secondary affordance to assess schizophrenia patients’ capacity to perceive affordances. Because of the documented decline across a wide range of cognitive domains in schizophrenia, the experiment was administered using a Go/No-Go protocol, a well-established procedure to assess decision-making in a wide variety of contexts, but simple enough to facilitate patients’ cooperation and completion of the task.

For the experiment, three pairs of mutually exclusive affordances were used: (a) *scoop-with/pierce-with*; (b) *pour-in-able/stretchable*; (c) *cut-able-with/mop-up-with*. Each affordance was represented by three objects sharing the same secondary affordance. Thus, six objects comprise one affordance pair (O_aff 1_, O_aff 1_) wherein O_aff 1_ had the first affordance (e.g., *scoop-with*) but not the second (e.g., *pierce-with*); O_aff 2_ had only the second affordance, but not the first. In each pair of affordances, one served as the target signal and the other as the distractor.

Schizophrenia patients were less accurate and slower than controls. However, when assessed for their capacity to detect the object’s physical properties (color, shape, material composition) in a control experiment, schizophrenia patients performed as accurately as controls and faster than they had in the affordance perception task. Based on these findings, the authors concluded that affordance perception capacity is likely impaired in patients with schizophrenia.

## Apraxia of Tool Use

Apraxia is a neurological disorder characterized by a marked impairment in performing skilled movements in response to a verbal command, despite intact sensory and motor abilities and comprehension of the task. Although apraxia is a predominant symptom in patients with left brain damage (LBD) after a cerebral vascular accident, it is also one of the core features of dementia and is included in diagnostic guidelines for AD. Recently, a growing number of studies have begun to explore the impact of AD on consciousness (Weiler et al., 2016; Bajic et al., 2021; Bomilcar et al., 2021). We now know that neuropsychiatric symptoms (e.g., depression, delusions and hallucination, agitation, and aggression) are common in AD. Whereas schizophrenia has garnered intense interest from phenomenology, little is known about the impact of AD on minimal self. Of interest, in this regard, is Pazzaglia and Galli (2014), who proposed that apraxia be considered as a disturbed sense of agency. Given that sense of agency constitutes one of the two defining features of minimal self, if (as Pazzaglia and Galli contend) sense of agency is disturbed in patients with apraxia, it is equally likely that patients’ perceptual capacity for affordances is disturbed as well.

One significant aspect of apraxia is that it affects a person’s ability to use commonly available tools or adapt other objects in the surrounding environment as tools to solve a given problem. Clearly, impaired ability to use tools will limit an individual’s functional capacity. Currently, two dominant competing hypotheses (manipulation-based and reasoning-based) try to account for variations in tool use behavior. The manipulation-based account relies on semantic memory, which is accumulated through prior sensorimotor experiences with a particular tool. In this view, an individual, when using a familiar tool, retrieves information from stored sensorimotor experiences (manipulation knowledge) about the tool’s purpose, its target object, and the typical movement associated with the tool. The reasoning-based account views the problem a potential tool user faces as an instance of problem-solving in which the individual uses mechanical knowledge to reason about the structural properties of tools and their action targets to solve the current problem.

Based on their literature review of studies examining tool use disorders in LBD patients, Baumard et al. (2014) concluded that failure of mechanical knowledge was the cause of the tool use deficit in LBD patients. Lesourd et al. (2016) investigated whether impaired tool use in AD is the same as in LBD. When given mechanical problem-solving tasks, AD patients (despite difficulties) engaged in strategies like trial-and-error to solve problems, a pattern not seen in LBD patients. The authors concluded that impairment of mechanical knowledge does not underlie tool use deficit in AD, but left open the question of the source of tool use impairment in these patients.

Proponents of the manipulation-based account of tool use suggest that, in cases involving novel tools, affordances may facilitate their applications. Kim et al. (2022) repeated the experiments conducted in Kim and Kim (2017) with four groups, AD, mild cognitive impairment (MCI), Parkinson’s disease (PD), and elderly controls (EC). The AD group performed poorest, followed by MCI, PD, and EC, in that order. EC and PD groups performed comparably. AD patients responded randomly to stimuli. MCI patients’ performance did not differ significantly from PD, EC, or AD groups, suggesting only a slight degradation in performance. In a control experiment in which participants were asked to report the physical properties of the same objects, all four groups performed reliably. These results provide preliminary evidence that affordance perception capacity is impaired in patients with AD and MCI.

## Discussion

When we open our eyes upon waking, many things arise in our fields of view, for example, a spouse, a pet, familiar furniture, and the layout of the room, with the additional smell of coffee aroma and the sound of the coffee maker gurgling from the kitchen. These things revitalize our mind and body. The orbits of the eyes, nose, cheek, upper lip, and limbs projected to the same location in our fields of view assure us that we are alive and (at least somewhat) ready to begin the day’s activities. For phenomenological philosophers, what is conveyed through our fields of view on opening our eyes is “mine-ness,” (i.e., recognition that we are the agent and the owner of our own actions, experiences, thoughts, and feelings). This is ipseity, i.e., the minimal self.

Upon opening our eyes, eye level immediately scales the surrounding layout and surfaces in units of “eye height” (Warren, 1984; Mark, 1987; Warren and Whang, 1987; Wraga, 1999). Partitioned in terms of eye-height, our surroundings reveal their various affordances (i.e., sit-on-able places, grasp-able objects, pass-able openings, drink-able liquids, edible foods, view-able displays, pet-able pets, etc.) for us to use as needed.

Phenomenological psychiatrists and philosophers have offered the ipseity disturbance hypothesis to account for the symptoms of schizophrenia, the most debilitating and most perplexing, of all mental disorders. The ipseity disturbance hypothesis assumes that two interdependent processes (hyper-reflexivity and diminished self-presence) disturb the minimal self, which in turn disturbs awareness of reality (one’s “grip” or “hold” on the world), eventually causing the patient to become disembodied and alienated from the surrounding world (Sass and Parnas, 2003; Fuchs, 2009).

To date, considerable effort has been devoted to further elucidate the phenomenology of self-disorders. Also drawing interest among phenomenological researchers is the search for the biological substrates of minimal self-disturbance in schizophrenia (Kyselo, 2016; Nelson and Sass, 2017; Nelson and Sass, 2017). Although their efforts to unpack seemingly incomprehensible utterances of patients are commendable, many puzzling questions remain, one of which is the connection between the disturbance of minimal self and the two processes underpinning the manifested symptoms of schizophrenia. Why does self-disturbance trigger these two processes? Conversely, why is the minimal self so susceptible to these two processes? Curiously, these issues have rarely been discussed by phenomenological researchers.

Setting these issues aside, we characterize what philosophers call the minimal self as the *ecological self*. Note that there are no clear criteria to define the minimal self except for some vague intuitive feeling of “a basic, immediate, or primitive “something” that we are willing to call a self” (Gallagher, 2000, p. 15). The ecological self, on the other hand, is defined based on an invariant pattern in the optical structure specific to it, that is, a global transformation of the optic array. Indeed, the specification of ecological self as a global transformation of optic flow was cleverly demonstrated by David Lee’s now classic “swinging room” research. Lee (Lee and Aronson, 1974; Lee and Lishman, 1975) constructed a room with a fixed floor, but with walls and ceiling that can be swung back and forth. When placed in this room and the walls moved, observers swayed in accordance with the optic flow pattern engendered by the moving room. This swaying occurred despite the fact that balance information (i.e., interoceptive information) provided by the inner ear and the receptors in the muscles and joints signaled that the observers’ postures were stationary.

Neisser (1993) credited the ecological self as the first form of self to develop in early infancy. To that extent, we construe ecological self as equivalent to minimal self. Thus, it is ecological self that is likely altered in schizophrenia. As in the case with the question pertaining to the particular symptoms manifested in schizophrenia, we have yet to determine how an altered ecological self would manifest. One plausible rationale might be that the locus of the ecological self may coincide with the locus of the observation point to which optical angles subtended by the observer’s body parts project. When a patient starts to experience alienation of the self from the body, it may be that the ecological self has separated (at least in part) from the observation point. As the ecological self gradually drifts away from the observation point, the geometric relationship between environmental objects and their corresponding optical angles is no longer preserved. Such disruption would have an immediate impact, distorting scale factors such as eye height, which would perturb the proprioceptive information specifying the ecological self.

For Gibson, perception of the environment and perception of self are co-implicative. Assuming Gibson is correct, if patients’ perceptions of self are disturbed, then, relatedly, their attunement to exteroceptive information (facts about the external environment) is likely to be disturbed, as well. With their perceptual capacity disturbed, these patients can no longer tune into the information specifying affordances. Consequently, their surroundings that once abounded with values and meanings become, for them, a barren field of value-neutral physical objects. We hypothesized that inability to appreciate the affordances comprising their surroundings might underlie the various symptoms manifested by schizophrenia patients. The results of Kim and Kim (2017) corroborated that hypothesis. Schizophrenia patients performed poorly when asked to identify unintended functions of human-made artifacts, but retained their capacity to identify physical properties (e.g., color, shape, and material composition) of the same objects.

Apraxia is defined as the inability to perform skilled actions despite intact sensory and motor abilities. To further explore the claim that apraxia is a manifestation of lost sense of agency (the key factor defining minimal self), Kim et al. (2022) administered the same task performed previously by schizophrenia patients to patients with AD. Patients with MCI, PD, and EC also participated in the study. The AD group performed poorest, followed by MCI, then PD and EC (differences for the latter two were not statistically different). The AD group responded randomly to stimuli, their performance not differing from chance. However, when asked to report the physical properties of the same objects, all four groups performed reliably.

Affordance remains a highly controversial concept. As a theoretical concept, most discussions of affordance have involved clarification of its ontological status. Warren’s (1984) seminal stair riser research led initial efforts to validate this concept empirically. Yet, as a concept founded on the principle of mutuality binding the reciprocal pairs of proprioception-exteroception, perception-action, animal-environment, and subjective-objective, designing a testing ground for an empirical validation of affordance comprehensive enough to encompass these aspects of dualities has been a challenge. We explored an indirect way to validate affordance by seeking contra-positive evidence.

In logic, the contra-positive of a conditional statement (if P, then Q) is formed by negating both antecedent and consequent and reversing them (if not Q, then not P) (where P stands for the antecedent and Q stands for the consequent). Thus, “If A, then B” is a direct proof, whereas, “If not B, then not A” is a proof by contra-positive, and these two are logically equivalent. Whereas the concept of affordance can be proven by direct evidence, we show how it can also be proven by contra-positive evidence.

As underscored above, affordances enable individuals to hold meaningful relationships with their surroundings. Those individuals whose capacity to tune into the information specifying affordances is somehow disturbed would be unable to detect affordances. Any dysfunction in affordance perception capacity would block access to meanings and values for these individuals, leaving only value-neutral physical objects. Imprisoned in meaningless surroundings, these individuals would be unable to keep in touch with their immediate environment. Eventually they become alienated, withdrawing from their surroundings and from other individuals. We reason that if one is capable of perceiving affordances, one can hold meaningful relationships with one’s surroundings and stay mentally healthy. By contra-positive logic, we can also reason conversely that, if one suffers from severe mental health symptoms and even withdraws from others and from one’s surroundings, one’s affordance perception capacity must be dysfunctional, depriving values and meanings from the individual, thus preventing the individual to keep in touch with one’s surroundings. We have described two studies conducted in our laboratory in which patients with schizophrenia (Kim and Kim, 2017) and AD (Kim et al., 2022) performed an affordance perception task. In both studies, the patients with schizophrenia performed poorly in comparison to healthy elderly controls and patients with other neurodegenerative disorders (e.g., PD). These results demonstrate a deficiency in affordance perception capacity that qualifies as contra-positive evidence for the concept of affordance.

So far, our discussion has focused on those patients with mental disorders whose capacity to perceive affordances has been severely impaired. In contrast, de Haan et al. (2013) studied people with Obsessive-Compulsive Disorder (OCD) whose symptoms were so severe that the only treatment available to them was deep brain stimulation (DBS). Much to everyone’s delight, the impact of DBS was remarkable, producing a profound change in patients’ experience of being in the world. Taking an eclectic position between enactivism and ecological psychology, the authors attempted to explain the phenomenological changes these patients experienced after DBS as a change in the field of relevant affordances. For example, if depicted as a 3D bar graph, the field of relevant affordances for normal individuals included bars of various heights and colors. For depressed patients, however, the field was shown as gray bars of the same short height to underscore how inconspicuous their surroundings were to them. For OCD patients, the field was depicted as a few tall and brightly colored bars to highlight the voracious consumption of their attention and obsessions.

Whereas de Haan et al. (2013) focused on characterizing patients’ experiences after DBS implantation in terms of the configuration of fields of affordances, ultimately the utilization of affordances hinges on the individual’s ability to register them. If the patient’s capacity to perceive affordances (i.e., the capacity to tune into the information specifying affordances) is impaired, despite how salient a particular affordance might be, the individual would not be able to realize it. Not having assessed DBS patients’ affordance perception capacity, as we did with patients with schizophrenia in Kim and Kim (2017) or AD in Kim et al. (2022), we cannot be definitive in our conclusions. However, we suspect that these patients would fit well into the same conceptual framework we used to explain the performance of the patients who participated in our studies. Specifically, we suggest that OCD likely impaired these patients’ capacity to perceive affordances, thus entrapping them in an environment with few affordances. DBS then restored the patients’ affordance perception capacity, enabling them to rejoice in the abundant affordances comprising their surroundings. For now, we remain curious as to whether the perceptual capacity to detect affordances of these OCD patients would have been similarly impaired as the patients with schizophrenia or AD were in our two studies (Kim and Kim, 2017; Kim et al., 2022).

In the two studies referred to above, we observed a substantial deficit in the capacity to perceive affordances for patients with schizophrenia and AD. With their perceptual capacity for detecting affordances impaired, these patients may find it difficult to keep in touch with their surroundings. Nevertheless, as demonstrated in these studies, these patients are still capable of detecting physical properties, suggesting that they should be able to manage contact with the surroundings to some extent. However, the kind of contact they can manage with the world becomes what Heft refers to as the “second-order mode of knowing.” To engage in this mode of knowing, Heft (2003) contends, one has to “step outside of the ongoing flow of immediate perception-action awareness by reflecting on the things of the environment; that is, [one has to] shift the necessarily selective character of [one’s] attentional focus from experiencing the immediate flow of events to experiencing the experience and, in doing so, isolate particular portions of immediate experience, holding them in awareness for analysis, categorization, or other second-order or indirect acts of cognition” (p. 151). Heft goes on to describe this mode: “accompanying these acts of reflexivity is a comparative heightening of awareness, as entities in experience are momentarily lifted out of the perceptual flow for closer scrutiny.” Heft’s description reminds us of hyper-reflexivity, one of the two characteristic processes disturbing patients with schizophrenia.

Taken together, these patients may be able to experience physical objects, but only as neutral things devoid of any psychological values. Not being able to relate to these objects (i.e., not being able to perceive affordances), they are not “drawn toward them or repelled by them for any intrinsic qualities they possess” (Heft, 2003, p. 151). Thus, these patients appear as if they are detached from the world.

## Conclusion

The standard account of (visual) perception starts with the light reflected from the surface of an object. Upon reaching sensory receptors, the light is converted to neural signals which then travel through various areas in the brain where they are embellished with the aid of the information stored in the memory. As purely mechanized responses to meaningless input signals, meaning is absent until semantic memory intervenes.

Meaning motivates animals. Consequently, animals are attracted to affordances that convey the meanings emerging from the objects with which they interact. Affordance is not just an important concept of a particular psychology theory but, as embodiments of meanings and values, it entails serious psychological implications. If an animal’s capacity to apprehend affordances is disabled, that animal would be deprived of objects’ meanings. Bereft of the motivation affordances offer, the animal may no longer engage with its surroundings. Eventually the animal would be alienated, both from itself and from the world, becoming a disembodied self or spiritless body (Fuchs, 2009, 2013).

To date, the search for direct evidence for affordance’s validity has been conducted primarily by assessing the capacity of healthy participants to perceive affordances [Warren (2021), for review].^1^ However, given the psychological values of affordance, a contra-positive statement can also qualify as valid. As noted earlier, a conditional statement (if P, then Q) can be formulated such that: if an individual perceives affordances (P), the individual comes in relationship with values and meanings, thereby maintaining meaningful relationships with the environment (Q). A contra-positive statement (if not Q, then not P) would be: If an individual is deprived of values and meanings, eventually experiencing severe mental suffering (not Q), the individual must have been unable to perceive affordances (not P).

This contra-positive statement appears to be true for patients with mental and clinical disorders that are presumed to be caused by disturbance of ipseity or self-disorder. In two studies conducted in our laboratory, we found that the capacity to perceive affordances was severely impaired in schizophrenia patients and AD patients, an existence proof corroborating affordance. Recently, some authors have contended that the ipseity disturbance (or self-disorder) model can extend to other mental disorders such as PTSD (Ataria and Horovitz, 2021), depersonalization disorder, and panic disorder (Sass et al., 2018). It is important to determine whether a similar decline in affordance perception capacity can be observed in these populations as in schizophrenics (Kim and Kim, 2017) and AD (Kim et al., 2022).

Affordances, when perceived, are used to regulate the action needed to attain an intended goal. Action, in turn, fine-tunes the perceptual system to be more sensitive to invariants specifying those affordances. Thus, perception and action are coupled cyclically until the intended goal is realized. When affordance perception capacity is disturbed, an observer may be unable to appreciate the rich meanings and values the surrounding environment offers. An affordance perception deficit can trigger a cascade of reactions that lead ultimately to the mental suffering seen in patients with schizophrenia and AD. Gibson (1982) admonished us that “the notion of affordances implies a new theory of meaning” (p. 409). Our findings support a strong argument for exploring the validity of affordance, in particular, and psychological reality, in general, from the perspective of values and meanings.

In conclusion, we suggest that contra-positive evidence of affordance can complement direct evidence for the concept. We suggest further that this integration can enable us to establish a stronger methodological foundation to design research that can help validate affordance empirically and further elucidate the psychological meaning embodied in affordance. In addition, based on our experience, we also suggest that clinical populations, particularly those arising from disturbance of minimal (i.e., ecological) self, can serve as fertile ground from which we can harvest contra-positive evidence, further corroborating Gibson’s concept of affordance.

## Author Contributions

Both authors contributed to manuscript from conception, drafting, revision, read, and approved the submitted version.

## Conflict of Interest

The authors declare that the research was conducted in the absence of any commercial or financial relationships that could be construed as a potential conflict of interest.

## Publisher’s Note

All claims expressed in this article are solely those of the authors and do not necessarily represent those of their affiliated organizations, or those of the publisher, the editors and the reviewers. Any product that may be evaluated in this article, or claim that may be made by its manufacturer, is not guaranteed or endorsed by the publisher.

## Footnotes

1 But see Pellicano et al. (2017) for attempts to elucidate apraxia deficits in terms of affordance mechanisms. See also Sevos et al. (2013), who demonstrated that patients with schizophrenia performed poorly in an affordance detection task; and Rounds and Humphreys et al. (2000), who attempted to rationalize limb apraxia arising from an abnormal sensitivity to competition in the presence of multiple affordances. We must note that our understanding of affordance differs from that of Rounds and Humphreys.

## References

[B1] Arango C. Carpenter W. T. (2011). “The schizophrenia construct: symptomatic presentation,” in *Schizophrenia*, 3rd Edn, eds Weinberger D. R. Harrison P. J. (Oxford: Wiley-Blackwell), 9–23.

[B2] Ataria Y. Horovitz O. (2021). The destructive nature of severe and ongoing trauma: Impairments in the minimal self. *Philos. Psychol.* 34 254–276. 10.1080/09515089.2020.1854709

[B3] Bajic V. Misic N. Stankovic I. Zaric B. Perry G. (2021). Alzheimer’s and consciousness: how much subjectivity is objective? *Neurosci. Insights* 16:26331055211033869. 10.1177/26331055211033869 34350401 PMC8295942

[B4] Baumard J. Osiurak F. Lesourd M. Le Gall D. (2014). Tool use disorders after left brain damage. *Front. Psychol.* 5:473. 10.3389/fpsyg.2014.00473 24904487 PMC4033127

[B5] Bomilcar I. Bertrand E. Morris R. G. Mograbi D. C. (2021). The seven selves of dementia. *Front. Psychiatry* 12:646050. 10.3389/fpsyt.2021.646050 34054604 PMC8160244

[B6] Cahill C. Frith C. D. (1996). “A cognitive basis for the signs and symptoms of schizophrenia,” in *Schizophrenia: A Neuropsychological Perspective*, eds Pantelis C. Nelson H. E. Barnes T. R. E. (New York, NY: John Wiley) 373–395.

[B7] Cermolacce M. Naudin J. Parnas J. (2007). The “minimal self” in psychopathology: re-examining the self disorders in the schizophrenia spectrum. *Conscious. Cogn.* 16 703–714. 10.1016/j.concog.2007.05.013 17632013

[B8] de Haan S. (2020). *Enactive Psychiatry.* Cambridge: Cambridge University Press.

[B9] de Haan S. Rietveld E. Stokhof M. Denys D. (2013). The phenomenology of deep brain stimulation-induced changes in OCD: an enactive affordance-based model. *Front. Hum. Neurosci.* 7:653. 10.3389/fnhum.2013.00653 24133438 PMC3794192

[B10] Fajen B. R. Kim N.-G. (2002). Perceiving curvilinear heading in the presence of moving objects. *J. Exp. Psychol. Hum. Percept. Perform.* 28 1100–1119. 10.1037//0096-1523.28.5.1100 12421058

[B11] Flament-Fultot M. Nie L. Carello C. (2016). Perception-action mutuality obviates mental construction. *Constr. Found.* 11 298–307.

[B12] Fuchs T. (2009). Embodied cognitive neuroscience and its consequences for psychiatry. *Poiesis Praxis* 6 219–233.

[B13] Fuchs T. (2010). The psychopathology of hyperreflexivity. *J. Specul. Philos.* 24 239–255.

[B14] Fuchs T. (2013). “The self in schizophrenia: jaspers, schneider, and beyond,” in *One Century of Karl Jaspers’ General Psychopathology*, eds Stanghellini G. Fuchs T. (Oxford: Oxford University Press), 245–257.

[B15] Gallagher S. (2000). Philosophical conceptions of the self: implications for cognitive science. *Trends Cogn. Sci.* 4 14–21. 10.1016/s1364-6613(99)01417-5 10637618

[B16] Gibson J. J. (1977). “The theory of affordances,” in *Perceiving, Acting, And Knowing: Toward an Ecological Psychology*, eds Shaw R. E. Bransford J. (Hillsdale, NJ: Lawrence Erlbaum Associates), 67–82.

[B17] Gibson J. J. (1979). *The Ecological Approach To Visual Perception.* Boston, MA: Houghton-Mifflin.

[B18] Gibson J. J. (1982). “Notes on affordances,” in *Reasons For Realism*, eds Reed E. Jones R. (Hillsdale, NJ: Lawrence Erlbaum Associates), 401–418.

[B19] Heft H. (2003). Affordances, dynamic experience, and challenge of reification. *Ecol. Psychol.* 15 149–180.

[B20] Heft H. (2020). Ecological psychology and enaction theory: divergent groundings. *Front. Psychol.* 11:991. 10.3389/fpsyg.2020.00991 32547449 PMC7271815

[B21] Humphreys G. W. Fode E. M. E. Francis D. (2000). “The organization of sequential actions,” in *Attention and Performance XVIII: Control of Cognitive Processes*, eds Monsell S. Driver J. (Cambridge, MA: MIT Press), 427–442.

[B22] Insel T. R. (2010). Rethinking schizophrenia. *Nature* 468 187–193. 21068826 10.1038/nature09552

[B23] Jablensky A. (2010). The diagnostic concept of schizophrenia: its history, evolution, and future prospects. *Dialogues Clin. Neurosci.* 12 271–287. 10.31887/DCNS.2010.12.3/ajablensky 20954425 PMC3181977

[B24] Kim N.-G. Kim H. (2017). Schizophrenia: an impairment in the capacity to perceive affordances. *Front. Psychol.* 8:1052. 10.3389/fpsyg.2017.01052 28701973 PMC5487489

[B25] Kim N.-G. Effken J. A. Lee H.-W. (2022). Impaired affordance perception as the basis of tool use deficiency in Alzheimer’s disease. *Healthcare* 10:839. 35627976 10.3390/healthcare10050839 PMC9140866

[B26] Kyselo M. (2016). The enactive approach and disorders of the self – the case of schizophrenia. *Phenomenol. Cogn. Sci.* 15 591–616. 10.1159/000369888 25720521

[B27] Lee D. N. Aronson E. (1974). Visual proprioceptive control of standing in human infants. *Percept. Psychophys.* 15 529–532. 10.3758/BF03199297

[B28] Lee D. N. Lishman J. R. (1975). Visual proprioceptive control of stance. *J. Hum. Mov. Stud.* 1 87–95.

[B29] Lesourd M. Baumard J. Jarry C. Etcharry-Bouyx F. Belliard S. Moreaud O. (2016). Mechanical problem-solving strategies in Alzheimer’s disease and semantic dementia. *Neuropsychology* 30 612–623. 10.1037/neu0000241 26523522

[B30] Mark L. S. (1987). Eyeheight-scaled information about affordances: a study of sitting and stair climbing. *J. Exp. Psychol. Hum. Percept. Perform.* 13 360–370. 10.1037//0096-1523.13.3.361 2958585

[B31] Neisser U. (1988). Five kinds of self-knowledge. *Philos. Psychol.* 1 35–59.

[B32] Neisser U. (1993). “The self perceived,” in *The Perceived Self*, ed. Neisser U. (Cambridge: Cambridge University Press), 3–21.

[B33] Nelson B. Sass L. A. (2017). Towards integrating phenomenology and neurocognition: possible neurocognitive correlates of basic self-disturbance in schizophrenia. *Curr. Probl. Psychiatry* 18 184–200. 10.1016/j.schres.2013.06.022 23810736

[B34] Nelson B. Parnas J. Sass L. A. (2014). Disturbance of minimal self (Ipseity) in schizophrenia: clarification and current status. *Schizophr. Bull.* 40 479–482. 10.1093/schbul/sbu034 24619534 PMC3984529

[B35] Noë A. (2004). *Action in Perception.* Cambridge, MA: MIT Press.

[B36] Parnas J. (2000). “The self and intentionality in the pre-psychotic stages of schizophrenia,” in *Exploring the Self: Philosophical and Psychopathological Perspectives on Self-Experience*, ed. Zahavi D. (Amsterdam: John Benjamins), 115–147. 10.1075/aicr.23.10par

[B37] Parnas J. (2003). “Self and schizophrenia: a phenomenological perspective,” in *The Self In Neuroscience And Psychiatry*, eds Kircher T. David A. (Cambridge: Cambridge University Press), 217–241.

[B38] Parnas J. (2012). The core gestalt of schizophrenia. *World Psychiatry* 11 67–69. 22654930 10.1016/j.wpsyc.2012.05.002 PMC3363374

[B39] Parnas J. Sass L. A. (2010). “Phenomenology of self-disorders,” in *The Embodied Self: Dimensions, Coherence And Disorders*, eds Fuchs T. Sattel H. C. Henningsen P. (Stuttgart: Schattauer), 227–244.

[B40] Pazzaglia M. Galli G. (2014). Loss of agency in apraxia. *Front. Hum. Neurosci.* 8:751. 10.3389/fnhum.2014.00751 25295000 PMC4172088

[B41] Pellicano A. Borghi A. M. Binkofski F. (2017). Editorial: bridging the theories of affordances and limb apraxia. *Front. Hum. Neurosci.* 11:148. 10.3389/fnhum.2017.00148 28424598 PMC5372806

[B42] Persons J. (1986). The advantages of studying psychological phenomena rather than psychiatric diagnoses. *Am. Psychol.* 41, 1252–1260. 10.1037/0003-066X.41.11.1252 3813184

[B43] Raballo A. Saebye D. Parnas J. (2011). Looking at the schizophrenia spectrum through the prism of self-disorders: an empirical study. *Schizophr. Bull.* 37 344–351. 10.1093/schbul/sbp056 19528205 PMC3044618

[B44] Read C. Szokolszky A. (2020). Ecological psychology and enactivism: perceptually-guided action vs. sensation-based enaction. *Front. Psychol.* 11:1270. 10.3389/fpsyg.2020.01270 32765330 PMC7381233

[B45] Reed E. S. (1996). *Encountering The World.* New York, NY: Oxford University Press.

[B46] Sass L. Borda J. P. Madeira L. Pienkos E. Nelson B. (2018). Varieties of self disorder: a bio-pheno-social model of Schizophrenia. *Schizophr. Bull.* 44, 720–727. 10.1093/schbul/sby001 29529266 PMC6007751

[B47] Sass L. A. (2003a). Negative symptoms, schizophrenia, and the self. *Int. J. Psychol. Ther.* 3 153–180.

[B48] Sass L. A. (2003b). “Self-disturbance in schizophrenia: hyperreflexivity and diminished self-affection,” in *The Self In Neuroscience and Psychiatry*, eds Kircher T. David A. (Cambridge: Cambridge University Press), 242–271. 10.1159/000488462

[B49] Sass L. A. (2014). Self-disturbance and schizophrenia: structure, specificity, pathogenesis (Current issues, New directions). *Schizophr. Res.* 152 5–11. 10.1016/j.schres.2013.05.017 23773296

[B50] Sass L. A. Parnas J. (2003). Schizophrenia, consciousness, and the self. *Schizophr. Bull.* 29 427–444. 14609238 10.1093/oxfordjournals.schbul.a007017

[B51] Sevos J. Grosselin A. Pellet J. Massoubre C. Brouillet D. (2013). Grasping the world: object-affordance effect in schizophrenia. *Schizophr. Res. Treat.* 2013:531938. 10.1155/2013/531938 24386567 PMC3872402

[B52] Sherrington C. S. (1906). *The Integrative Action Of The Nervous System.* New Haven, CT: Yale University Press.

[B53] Stanghellini G. (2009). Embodiment and schizophrenia. *World Psychiatry* 8 56–59. 19293962 10.1002/j.2051-5545.2009.tb00212.x PMC2652898

[B54] Varela F. Thompson E. Rosch E. (1991). *The Embodied Mind: Cognitive Science and Human Experience.* Cambridge, MA: Oxford University Press.

[B55] von Fieandt K. Gibson J. J. (1959). The sensitivity of the eye to two kinds of continuous transformation of a shadow-pattern. *J. Exp. Psychol.* 57 344–347. 10.1037/h0046028 13654647

[B56] Warren W. H. (1984). Perceiving affordances: Visual guidance of stair climbing. *J. Exp. Psychol. Hum. Percept. Perform.* 10 683–703. 10.1037//0096-1523.10.5.683 6238127

[B57] Warren W. H. (1998). Visually controlled locomotion: 40 years later. *Ecol. Psychol.* 10 177–219.

[B58] Warren W. H. (2006). The dynamics of perception and action. *Psychol. Rev.* 113 358–389. 16637765 10.1037/0033-295X.113.2.358

[B59] Warren W. H. (2021). Information is where you find it: perception as an ecologically well-posed problem. *I Perception* 12 1–24. 10.1177/20416695211000366 PMC7995459 33815740

[B60] Warren W. H. Whang S. (1987). Visual guidance of walking through apertures: body scaled information for affordances. *J. Exp. Psychol. Hum. Percept. Perform.* 13 371–383. 10.1037//0096-1523.13.3.371 2958586

[B61] Weiler M. Northoff G. Damasceno B. P. Balthazar M. L. F. (2016). Self, cortical midline structures and the resting state: implications for Alzheimer’s disease. *Neurosci. Biobehav. Rev.* 68 245–255. 10.1016/j.neubiorev.2016.05.028 27235083

[B62] Weinberger D. R. Harrison P. J. (2011). *Schizophrenia*, 3rd Edn. West Sussex: John Wiley & Sons.

[B63] Wong A. H. C. Van Tol H. H. M. (2003). Schizophrenia: from phenomenology to neurobiology. *Neurosci. Biobehav. Rev.* 27 269–306. 12788337 10.1016/s0149-7634(03)00035-6

[B64] Wraga M. (1999). The role of eye height in perceiving affordances and object dimensions. *Percept. Psychophys.* 61 490–507. 10.3758/bf03211968 10334096

[B65] Zahavi D. (2005). *Subjectivity And Selfhood: Investigating The First-Person Perspective.* Cambridge, MA: MIT Press.

